# The 1.06 frequency ratio in the cochlea: evidence and outlook for a natural musical semitone

**DOI:** 10.7717/peerj.4192

**Published:** 2017-12-21

**Authors:** Andrew Bell, W. Wiktor Jedrzejczak

**Affiliations:** 1 John Curtin School of Medical Research, Australian National University, Canberra, Australian Capital Territory, Australia; 2 Institute of Physiology and Pathology of Hearing, Warsaw, Poland; 3 World Hearing Center, Kajetany, Poland

**Keywords:** Human cochlea, Spontaneous otoacoustic emission, Musical perception, Semitone, Music

## Abstract

A frequency ratio of about 1.06 often appears in cochlear mechanics, and the question naturally arises, why? The ratio is close to that of the semitone (1.059) in music, giving reason to think that this aspect of musical perception might have a cochlear basis. Here, data on synchronised spontaneous otoacoustic emissions is presented, and a clustering of ratios between 1.05 and 1.07 is found with a peak at 1.063 ± 0.005. These findings reinforce what has been found from previous sources, which are reviewed and placed alongside the present work. The review establishes that a peak in the vicinity of 1.06 has often been found in human cochlear data. Several possible cochlear models for explaining the findings are described. Irrespective of which model is selected, the fact remains that the cochlea itself appears to be the origin of a ratio remarkably close to an equal-tempered musical semitone, and this close coincidence leads to the suggestion that the inner ear may play a role in constructing a natural theory of music. The outlook for such an enterprise is surveyed.

## Introduction

Our understanding of the cochlea has increased enormously since 1978 when Kemp reported that the inner ear produced faint echoes in response to a click (Kemp, 1978). These otoacoustic emissions (OAEs) have shown that the human cochlea is an active detector of sound, not just a passive microphone (Bell, 2004), just like Gold suspected nearly 70 years ago (Gold, 1948). Most convincingly, the cochlea even emits continuous, pure tones without stimulation (spontaneous otoacoustic emissions, SOAEs), striking evidence of its active status (Zurek, 1981).

OAEs have enabled us to noninvasively probe the inner workings of the cochlea, providing accurate, fine-grain data on the hearing organ’s fine frequency resolution (Shera & Abdala, 2012). Based on both psychophysical and otoacoustic studies, the tuning elements in the human cochlea have been measured to have effective *Q* values of about 10–30 over the range 1–10 kHz (Shera, Guinan & Oxenham, 2002), indicating a highly tuned condition. Accordingly, the suggestion has been made that the cochlea might be considered to operate along resonance principles rather than in terms of the usual traveling wave models (Bell, 2012).

Whatever the most appropriate model may be, one of the most interesting findings to emerge from OAE studies is that the most-common ratio between neighbouring emission frequencies occurs at an interval of about 1.06, although there is a distribution in values which ranges from 1.04 to 1.08 and greater. The peak in the ratio is notable in that it has musical significance. The semitone in music is the smallest interval in Western musical scales (Burns & Ward, 1982; Parncutt, 1989), so that 12 semitones together comprise an octave (frequency ratio of 2:1). A similar ratio appears in many non-Western scales, although by itself it appears absent from Chinese, Arabian, and Javanese musical forms (Ball, 2010). Confining ourselves to Western music for now, the semitone is equal to 2^(1/12)^ or 1.059 on the equal tempered scale or 16/15 (1.067) in Just intonation. The presence in the cochlea of an approximation to a semitone has been pointed out by several researchers (Bell, 2002; Blinowska et al., 2012; Braun, 1997; Braun, 2006; Braun, 2013), but in general the musical link has not been widely recognised. Perhaps the most-frequent ratio is merely coincidence (D’Amato, 1988; Justus & Hutsler, 2005)?

This paper investigates the closest neighbour issue in more detail, and presents further evidence from OAEs that there is a preferred frequency ratio of 1.06 ± 0.005. Prima facie, this suggests the human cochlea may be naturally configured to detect semitone intervals, and by extension perhaps other musically significant ratios. Such innate musicality would be startling, but a ‘natural’ basis for music might explain the powerful musical tendencies of all human cultures and our ability to instantly recognise musical intervals. Even young babies can recognise melodies and harmonies, an ability which has been difficult to explain on the view that music is simply a matter of culturally acquired familiarity (McDermott & Hauser, 2005). Since the time of Pythagoras, many have claimed that music has some sort of naturalistic origin, but usually these have been cast in terms of acoustical physics or neural circuitry. In the discussion section of this paper, the main issues surrounding a natural theory of music are canvassed, and an assessment is made of whether a natural cochlear semitone might play a useful role in such a theory.

First, however, we make a general survey of findings where a frequency ratio close to a semitone has been found in OAE data from the human cochlea. Small ratios have also been found in animal data too, but these findings are not covered here. In general, the ratios in laboratory animals are somewhat larger than found in humans, but of course such findings are not directly relevant to human musical perception.

### Ratios in SOAE data

The first work to find regularity in the spacing of OAEs was that done by Dallmayr more than 30 years ago (Dallmayr, 1985; Dallmayr, 1987). He studied both spontaneous and stimulus frequency OAEs in populations of human subjects (119 and 15 respectively) and found that a histogram of the intervals between neighbouring emissions peaked at 0.35–0.4 Bark, where the Bark is a psychophysical measure used to measure the relative bandwidth of a sound. The Bark scale varies somewhat with frequency, but in the 2 octaves between 1 and 4 kHz there are 9 Bark, meaning that 1 Bark is about 0.22 octave and 0.35 Bark is 0.08 (one-twelfth) of an octave, practically a semitone. In work with SFOAEs (Dallmayr, 1987), regular spacing of maxima and minima were found, typically occurring at the same frequencies as SOAEs. This time, a histogram of spacings showed a peak at 0.3–0.35 Bark, slightly smaller than a semitone (falling between 1/13 to 1/15 of an octave).

The first to point out the musical significance of SOAEs was Braun, who pooled SOAE data from all available sources and examined their separation (not necessarily nearest-neighbour) in terms of cents, where 100 cents = 1 equal-tempered semitone (Braun, 1997). When the data were analysed into 11-cent bins and given a 3-point smoothing, the distribution peaked at 100 ± 5 cents, both for males and females. Although there were some irregularities in the distribution at larger ratios, after smoothing there was no other outstanding ratio. A later paper (Braun, 1998) is of interest in comparing ratios of SOAEs between the two ears, and one distinctive finding was a statistically significant tendency for SOAEs to be present at the same frequency in both ears of the same subject, a property Braun called ‘mirroring’. However, the effect was found to be weak, and no other significant ratios were found.

De Kleine et al. (2000) reported SOAE frequencies for one subject who had six SOAEs at 1,268, 1,348, 1,430, 1,525, 1,612, and 1,720 Hz. The ratios between these frequencies are 1.06, 1.06, 1.07, 1.06, and 1.07, giving a geometric mean of 1.063, very close to the equal-tempered semitone of 1.059 and the Just semitone of 1.067. Other data from De Kleine et al. (2000) show a regular run of 9 SOAEs varying from 979 Hz to 1,848 Hz, from which a geometric mean ratio of 1.073 (122 cents or one-tenth of an octave) can be calculated.

Braun (2006) reported further analysis of SOAEs which revealed another interesting property with musical implications. When SOAE data from both children and adults was analysed, it was found that there was a bimodal distribution, with a population peak at 1.5 kHz and 3.0 kHz (although the two peaks were absent in neonates). Comparing different data sets and shifting them progressively by 7 to 17 semitones, Braun found that a maximum correlation occurred at a shift of 12 semitones (1 octave), suggesting another possible musical ratio in OAE data.

Finally, an analysis by Braun (2013) of SOAEs from four subjects who had an unusually large number of emissions (168 in total) again confirms that the preferred ratio between neighbouring SOAEs is a semitone (100 ± 5 cents). Data from subject BD (Fig. 2 of Braun, 2013) clearly illustrate how the average ratio is very close to a semitone (horizontal lines in the plot).

### Ratios in transiently evoked OAEs

A method for extracting “resonant modes” from OAEs evoked by tone-bursts or clicks was developed by Blinowska and colleagues, a technique which uses a matching pursuit algorithm to identify waveforms of specific frequency in the cochlear responses (Blinowska, Jedrzejczak & Konopka, 2005; Blinowska, Jedrzejczak & Konopka, 2007; Jedrzejczak, Blinowska & Konopka, 2006). A remarkable property identified in continued application of this method was the identification of what appeared to be integer ratios between resonant modes. Applying the same method as before, some ratios between identified long-lasting echoes appeared to have integer ratios. In these cases the identified cochlear echoes appeared to contain musical ratios which included not only the semitone but also close approximations to most of the ratios comprising the 12-tone Just scale (Blinowska et al., 2012).

The Blinowska et al. (2012) paper analyses examples of sustained cochlear echoes from 86 ears and, based on this data, makes the surprising claim that the human cochlea is intrinsically musical. So far, the claim has not been independently substantiated. Certain theoretical and experimental reasons may be put forward for this. Theoretically, the model used to support the claim is based on the initial non-dispersive model of Bell (2002), without including the dispersive properties which have now been suggested by later work (Bell & Fletcher, 2004; Bell & Maddess, 2009). On the experimental side, the findings are susceptible to artefacts generated by harmonic distortion, which has the result of weakening the claims. While the semitone seems to be unambiguously present in the data, the other musical ratios might have arisen from a methodology in which all the detected echoes, no matter what their absolute frequency, were shifted up and down into a single 1-octave frequency range. In such an arrangement, any distortion falling in higher or lower octaves would be identified as a musical ratio. Nevertheless, the idea that certain core elements of music could originate from within the cochlea itself is intriguing, and warrants further investigation. The current paper provides more evidence: it shows that, when the cochlea is stimulated by clicks, the long-lasting echoes coming back contain a distribution of frequency ratios whose peak occurs very close to a semitone.

## Aim of the Study

The present study focuses on analysing the ratios between synchronized SOAEs (SSOAEs) in a group of 81 normally hearing young adults. In addition, some SOAE data from an earlier study is presented in which two bistable emissions alternately switched frequencies, and this unusual and rather rare phenomenon shows that the ratio between the two switching frequencies is 1.0600 ± 0.0005. Some relevant cochlear modelling efforts are then described which attempt to place the findings within the framework of a single physical model, and an assessment is then made of whether the findings could support the proposal that the human musical sense originates within the cochlea itself.

## Materials and Method

Measurements were performed on a group of normally hearing adults: 81 subjects (140 ears, 62 left, 78 right) of age 19–31 years. All subjects underwent pure tone audiometry, tympanometry, and OAE measurement. All had pure tone thresholds better than 25 dB HL over 0.5–8 kHz, type A tympanograms, and no known history of otologic disease. The subjects gave written informed consent prior to participation. Research procedures were approved by the Ethics Committee of the Institute of Physiology and Pathology of Hearing, Poland (IFPS/KB/06/2012).

**Figure 1:**
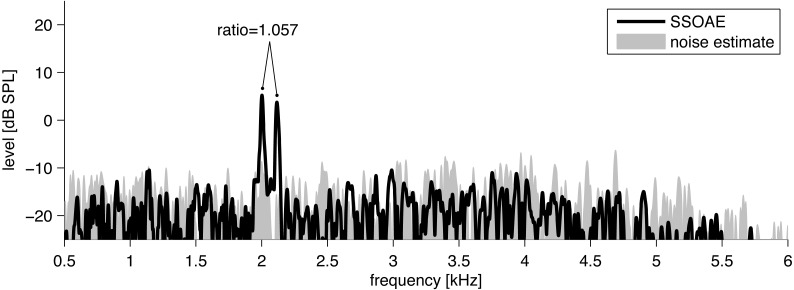
Example of the spectrum (black line) of click-evoked sound recorded from the ear canal of one subject. Two SSOAEs appear above the noise floor at 2,002 Hz and 2,116 Hz. The ratio in this case is 1.057.

OAEs were measured under low-noise ambient conditions using an ILO 292 system (Otodynamics Ltd., Hatfield, UK). Standard equipment protocols were used for measurement of synchronized SOAEs (SSOAEs). Click stimuli of amplitude 80 ± 3 dB peak SPL were used to evoke a total of 260 responses in each ear. SSOAEs were acquired under the linear protocol using a train of clicks (12.5 s^−1^) to synchronize SOAEs. SSOAEs were studied over the range 0.5–6 kHz, with frequencies accurate to ±6 Hz. The detection method is described below, and an example of SSOAEs acquired by the technique is shown in Fig. 1.

To detect SSOAEs, the following technique was used. SSOAEs were measured with an inter-stimulus interval of 80 ms, but of prime interest was the 20 ms window beyond 60 ms when most of the evoked components had faded away. In previous work with the ILO system, SSOAEs have usually been assessed visually by looking for spectral peaks in this 20 ms window which were 3 dB above the noise (Lamprecht-Dinnesen et al., 1998; Morlet et al., 1995). SSOAEs generally coincide in frequency with SOAEs measured without any stimulus (Wable & Collet, 1994). Here, because the dataset was large, SSOAEs were analyzed automatically by a time–frequency technique based on the matching pursuit (MP) algorithm (Mallat & Zhang, 1993) but using a slight modification to better account for the character of OAE components (Jedrzejczak et al., 2016; Jedrzejczak et al., 2009). The modified MP method decomposes SSOAE signals into discrete waveforms of defined frequency, latency, duration, and amplitude. Each waveform was compared to the background noise in a ± 100 Hz band around its centre frequency, and if its level exceeded the noise by 3 dB it was taken to be an SSOAE (Penner, Glotzbach & Huang, 1993; Ugur et al., 2009).

For measurement of bistable SOAEs, data from a previous study (Bell, 1992) was used and the equipment and methods are identical to descriptions given in that work. In brief, a Bruel & Kjaer 4179 low-noise microphone was coupled via flexible tubing to the ear of a subject who was seated comfortably in a sound-proof room; the signal was amplified, filtered, and recorded for later time–frequency analysis with a Hewlett-Packard 3582A spectrum analyzer.

Most analyses were done using the Matlab R2016A (Mathworks, Natick, MA, USA) package.

## Results

### SSOAEs

**Figure 2:**
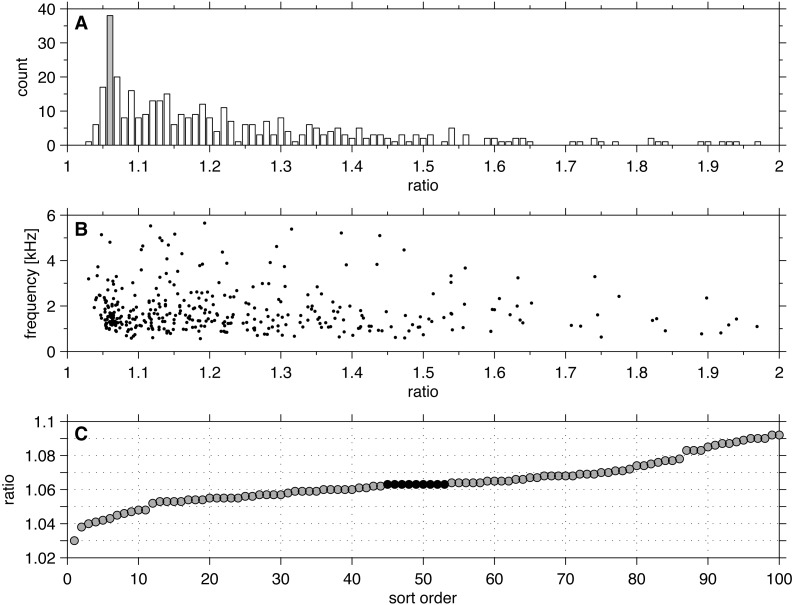
(A) Histogram of 394 ratios between pairs of neighbouring SSOAEs recorded from 140 ears of young adults. The peak appears in the bin 1.055 to 1.065 (shaded). (B) The ratios plotted against the lower frequency of the pair. (C) The ratios sorted in order of size and the first 100 plotted. The most prevalent ratio (9 points) appears at 1.063 (black).

The results of analysing the ratios between all neighbouring SSOAEs from 140 ears of 81 adults are shown in Fig. 2A. The distribution clustered around a distinct peak at 1.06. Of the 394 ratios, 63 (16%) fell within the interval 1.05–1.07, with 38 of them (10%) in the 1.06 bin (ratios 1.056–1.065).

Figure 1B shows that the peak at 1.06 occurs at frequencies over the full measured range, and there is no preference for it to occur at a particular frequency. Figure 1C shows a high resolution plot of all the ratios in the 1.06 region after sorting for size, and shows that the most prevalent ratio, after rounding to three decimal places, occurred at 1.063. There were nine SSOAEs which had this ratio, about twice as many as ratios in adjoining 0.001 bins, suggesting that the favoured ratio is 1.063 ± 0.001. The favoured ratio represents a musical interval of 106 ± 2 cents. The analysis confirms that SSOAEs share the same preference for the 1.06 ratio as SOAEs have been found to exhibit.

### Bistable emissions

**Figure 3:**
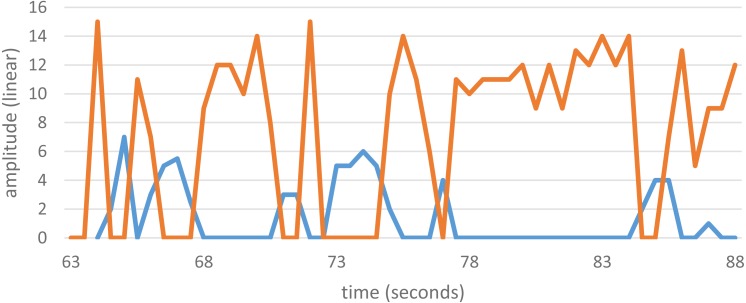
Amplitude variations of a bistable SOAEs. The two SOAEs exchange energy with each other so that only one is strongly active at any one time. The SOAEs have frequencies of 2,165.5 ± 0.3 Hz (blue) and 2,295.5 ± 0.3 Hz (orange), a ratio of 1.0600 ± 0.0005. Unpublished data from Bell (2005), available as Data S2.

Some SOAEs are bistable, meaning that energy swaps back and forth between two of them. Each state has its own distinct frequency, and the energy alternates every second or so. Bistable emissions are sometimes encountered in OAE studies, although they are rare, and data from a previous study (Bell, 1992) showing such behaviour is shown in Fig. 3. The bistable emission has frequencies of 2,165.5 ± 0.3 Hz and 2,295.5 ± 0.3 Hz, a ratio of 1.0600 ± 0.0005. Other examples of bistable emissions reported in the literature are also given in Table 1. The table shows that the frequency ratio ranges from 1.05 to 1.09.

**Table 1:** Examples of bistable SOAEs.

*f*1 (Hz)	*f*2 (Hz)	Ratio	Source
2,165.5 ± 0.3	2,295.5 ± 0.3	1.0600 ± 0.0005	This paper
1,378	1,443	1.047	Burns & Keefe (1992)
4,116	4,303	1.045	Burns & Keefe (1992)
1,612	1,700	1.055	Wit (1990)
1,595	1,700	1.066	Burns et al. (1984)
1,330	1,410	1.060	Burns et al. (1984)

## Discussion

Evidence has been assembled which indicates that frequency ratios of 1.05–1.07, with a peak very close to a semitone (1.060 ± 0.005), are widespread in the cochlear mechanics literature, a finding that has implications for the theoretical underpinnings of music. One possibility, discussed more fully later, is that the observed semitone could be identified as part of a natural theory of music in which the cochlea is able to instantly detect musical ratios. Although such an idea is speculative, there are findings in the literature which give it support. The significance here is a reduction in the amount of neural processing required to recognise a musical ratio, relieving the nervous system, and the brain in general, of perceptual effort (Zatorre & Salimpoor, 2013).

The proposal for an innate cochlear semitone aligns with evidence that music has both harmonic and melodic dimensions (Demany & Semal, 1990). More specifically, that the 1.06 ratio detected in the present work may reflect the operation of a hypothetical “automatic frequency-shift detector” (Demany & Ramos, 2005), based on semitones, built into the ear. The conjecture made here is that music is a natural property of the human ear, and a description of how this might work in terms of a physical model is set out below.

First, however, a brief survey of the approaches so far used in trying to understand the 1.06 ratio is made. All these perspectives involve different models of cochlear mechanics, and in general the musical implications of the ratio have not been emphasised. The models can be classified under four main headings.

### Multiple internal reflection

The most common explanation for the 1.06 ratio comes from the multiple internal reflection (MIR) model of Shera and Zweig which explains the ratio in terms of reflection of the traveling wave between the stapes and its peak (Shera & Guinan, 2008). This mechanism sets up a pattern of recirculating echoes which together creates a longitudinal standing wave and a narrowed response peak. Because a standing wave requires an integer number of wavelengths, and there are calculated to be about 15 wavelengths in the standing wave cavity, the conditions specify that the standing waves must have optimum wavelength ratios of 16/15 or 15/14 (Shera, 2003; Zweig & Shera, 1995), and these fractions could give rise to the favoured 1.06 frequency ratio. The MIR model accommodates the earlier work of Zweig (1991) and Shera (2001), where the cochlea was described in terms of closely spaced poles of an electrical network, with the poles occurring at two frequencies separated by a ratio of 1.06.

**Figure 4:**
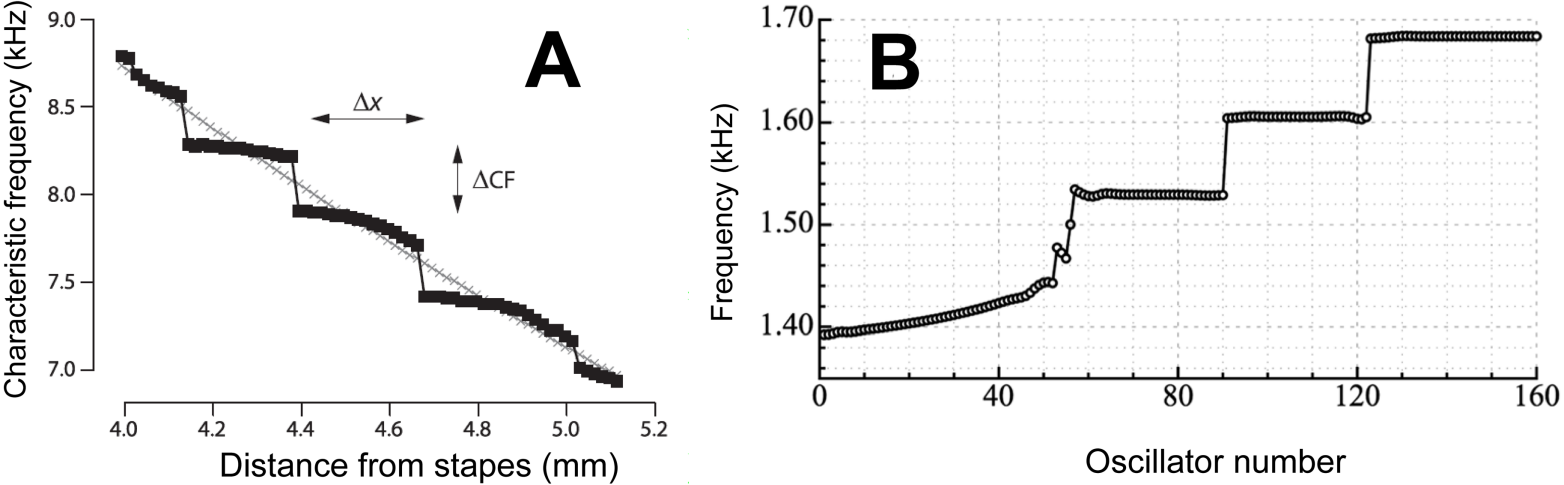
Staircases in the cochlea. (A) A staircase arrangement in the cochlea as derived by Shera using his multiple internal reflection model. Due to the reflection of waves at the two ends of the cochlea, steps in frequency (ΔCF) are produced which have a step ratio of about 1.05. From Shera (2015) with permission of the author. (B) To simulate human spontaneous otoacoustic emissions, Wit & Van Dijk (2012) modelled the elastic coupling of 160 oscillators uniformly graded in frequency from 1.4 to 1.7 kHz. The result of the coupling was that the oscillators clustered into a discrete set of frequency plateaus. The size of the frequency steps was about 1.05, although it depended on the strength of the coupling. Reprinted with permission of the Acoustical Society of America. Copyright 2012, Acoustic Society of America.

The MIR model has been developed into what Shera calls “the spiral staircase” (Shera, 2015) where the iterated reflections give rise to discrete groupings in the cochlea’s frequency response, and it is these groupings which produce a staircase-like sequence of frequency steps separated by about 1.06 (see Fig. 4A). The model is also compatible with the work of Zweig (2015) who, based on the experimental findings of Rhode, described in mathematical detail the mechanics and frequency response of the cochlea. A noteworthy feature of Zweig’s calculations is the appearance of two distinct frequency peaks: the main traveling wave peak and a smaller subsidiary peak located just beyond it. The frequency ratio between the two peaks is, based on Rhode’s monkey data, about 1.06. Zweig emphasises the notch between the two peaks, and says that explaining this notch—which he regards as a deep puzzle—is important for a full understanding of cochlear mechanics.

### Gammatone filterbanks

Most models of the cochlea emphasise the role of slow traveling waves in the cochlea rather than the direct effect of fast acoustic pressure waves (Puria & Steele, 2008). However, the possibility that there might be fast pressure-wave interactions within the cochlea (Recio-Spinoso & Rhode, 2015), perhaps from a direct effect on outer hair cells (Bell, 2008), provides stronger support to extensive earlier modelling efforts which used gammatones to replicate the dynamics of the cochlea (Lopez-Poveda, 2005; Lyon, 1998). The cochlea responds to sound in a similar way to that of a gammatone filter, so the idea of modelling the cochlea as a parallel bank of gammatone filters has had a long history (Lyon, 1996). However, a parallel filterbank takes simultaneous inputs, and for a long time this was seen as a drawback because of an apparent inconsistency with traveling wave models which call for sequential excitation of the cochlea’s tuned elements. If a fast mechanism for outer hair cell stimulation is possible, then the gammatone filterbank takes on greater relevance.

Of particular interest is the previous work with “dual resonance” models which combine two processing stages separated by a frequency ratio of 1.06. Based on an original idea of Goldstein (1990), such models use a combination of linear and nonlinear filters to simulate cochlear responses (Lopez-Najera, Lopez-Poveda & Meddis, 2007; Lopez-Poveda & Meddis, 2001). A distinctive feature is that the ratio between the modelled filters is typically chosen to be approximately a semitone; for example, Table 1 of Lopez-Poveda & Meddis (2001) shows ratios in the filterbank of 1.06, 1.09, 1.06, 1.06, 1.03, and 1.07.

### Coupled oscillators

The cochlea has most commonly been modelled as an electrical transmission line which supports a traveling wave of displacement that bends the stereocilia of hair cells (Reichenbach & Hudspeth, 2014). An alternative description is to consider the cochlea at its most basic as a chain of coupled oscillators, and leave aside the actual activation mechanism (Duke & Jülicher, 2003; Wit & Van Dijk, 2012). In this way, the coupled oscillator model concentrates on local dynamics rather than on global ones, and opens up possibilities for fast, long-range interactions as well as between neighbouring elements (Bell & Wit, 2015). In the cited work, the authors examined the result of simultaneously exciting all the cochlear resonators by using the classical model of the vibrating reed frequency meter. In this approach, the cochlea’s tuned elements were represented by coupled vibrating reeds and the reeds were driven all at once by an oscillating force. This resonance-style model reproduced a traveling wave, but of particular interest it also showed a main response peak and a secondary peak separated by a ratio of about 1.06. The paper shows that the actual size of the ratio depends on the strength of the elastic coupling between the reeds. Coupling between the reeds—taken to represent the cochlea’s tuned elements on the basilar membrane—is therefore an important factor, although it is usually ignored in traveling wave models (Nobili, Mammano & Ashmore, 1998). Coupling of reeds was also responsible for producing a second interesting feature: a sequence of minor peaks separated by a ratio of about 1.03, a corrugation of the system which might explain quasi-periodic ripples in fine-grain audiograms (Elliott, 1958), the use of quarter-tone tunings (the enharmonic diesis) in ancient musical genres (Pesic, 2010), and the regular phase rotation of stimulus frequency otoacoustic emissions (Shera, 2003). The paper of Bell & Wit (2015) goes on to show that the regular peaks are due to “frequency plateaus” in which oscillating elements cluster together into synchronised groups (Ermentrout & Kopell, 1984). The evidence presented in Braun (2013) also appears to support the coupled oscillator model.

These frequency plateaus were modelled in detail by Wit & Van Dijk (2012), and an example of their findings are shown in Fig. 4B, where again staircase-like structure in the cochlea arose. Modelling showed that when a chain of oscillators, graded in frequency from high to low are coupled together, the oscillators are forced into a series of quantised frequency steps, a phenomenon which, as the authors point out, has implications for the cochlea—since it too can be regarded as a coupled chain of tuned elements. The steps in this case resemble the steps in the staircase identified by Shera—they have the same size of between 1.05 and 1.10—even though the authors put forward different mechanisms to explain them.

### Feedback between outer hair cells

A model of the cochlea developed by Bell (2002) describes how SOAEs might be generated in terms of positive feedback of wavefronts between the rows of outer hair cells. This surface acoustic wave (SAW) model relies upon the active nature of outer hair cells (they are both sensors and motors) and the remarkably regular way in which outer hair cells are arranged. There are some intriguing properties arising from this geometry, notable here being a 19° angle between adjoining cells—commonly observed in micrographs of the human cochlea (Bell, 2005)—which might mean that feedback between the cells could produce musical ratios (in particular, the semitone would correspond to a ratio of intercellular distances of cos 19° = 1/1.06). However, at this point the SAW model is theoretical and requires further experimental support, and so detailed consideration is put aside pending future work.

### Assimilating OAEs and music

The preceding survey has indicated that there may be at least two or three physical mechanisms for the origin of the 1.06 ratio in the cochlea, and more work is needed in order to decide which model gives the best account. Some recent work has highlighted the role of elastic coupling along the basilar membrane (Wit & Bell, 2017), and the rest of this section will be concerned with establishing how this property can provide a direct explanation for the appearance of the 1.06 ratio, although of course it may only be part of the answer. The modelling work of Wit and Bell showed that each plateau in a chain of coupled oscillators behaved in a similar way to that of a single oscillator (at least so far as entrainment to external tones is concerned), an inference being that the 1.06 ratio might also arise from interaction between neighbouring steps of the cochlear staircase. In this way, each plateau in the cochlea might act as if it were a single oscillator, and there would be a ratio of 1.06 between adjoining oscillators.

Following through with this coupled oscillator model, the 1.06 interval between OAEs appears because a particular site on the basilar membrane has excess cochlear amplifier gain, generating a spontaneous emission at a corresponding frequency. This local oscillation will in turn entrain neighbouring oscillators, meaning that all the oscillators within a step will oscillate together. The dynamics of a coupled chain mean that if the driving oscillator is near the edge of a step, it may sometimes jump to, and entrain, oscillators on a neighbouring step, and a switching of frequencies will take place (Fig. 3). Evidently, the coupling strength between oscillators must be such that a natural semitone of about 1.06 is produced (Fig. 2).

The coupled oscillator picture suggests that the cochlea possesses innately tuned resonant elements, and that when these structures are stimulated acoustically, the resulting echoes contain a clear signature of a musically tuned array. The tuned elements might be part of a semitone-detection process related to the automatic pitch-detection mechanism described by Demany and colleagues. Such a mechanism would permit melodies to be identified with minimal neural processing.

Whatever the full scenario may be, the evidence suggests there is at least one aspect of the cochlea itself—and not the brain (as usually assumed)—which is innately musical. This is an intriguing possibility, and is an aspect which calls for closer investigation. The following sections outline evidence from the literature which supports the proposal that the cochlear semitone might be a building block within a natural theory of music.

### Evidence for a musical cochlea

Music is both beautiful and enigmatic. We know many details of how it works, but the bigger picture of why humans experience music in its unique shape and form—most distinctively, why there should be 12 semitones to the octave as found in many cultures—has been elusive (Ball, 2010; McDermott & Hauser, 2005). At this point, the first steps in bringing together the physiological and the psychological aspects of music is undertaken.

Fundamentally, there are two separate but linked aspects of music—harmony and melody Demany & Semal (1990)—and the suggestion is made that each aspect can be identified with a separate analysis mechanism in the cochlea. The harmonic template, first of all, could perhaps be based on some envelope detection process which depends on the way harmonics sum together, whereas, in a complementary fashion, the melodic template might involve detection of discrete steps along the basilar membrane—frequency plateaus separated by a ratio of 1.06 (the natural semitone). There are two distinct pieces of evidence supporting the dual template.

#### Enlarged octave

First, consider the curious case of the enlarged octave. The octave is the mainstay of music, and in Pythagorean terms it is a frequency ratio of precisely 2:1. Remarkably, however, this is not always experimentally true. Whereas in harmonic terms it is always possible to tune an instrument exactly to an octave by reducing the rate of beating of a fundamental and its first partial to zero, in melodic terms things are slightly different. Psychophysical experiments involving the fine-scale setting of two tones reveal that the octave is almost invariably ‘stretched’ (Hartmann, 1993; McKinney & Delgutte, 1999; Van den Brink, 1977). In general, the octave turns out to be about 20 cents higher than 2:1, although the stretch depends on the subject and the frequency. Typically, the ratio turns out to be about 2.03:1 (McKinney & Delgutte, 1999, Fig. 1). Reinforcing the case for a natural semitone, the twelfth-root of 2.03 is 1.061, and this ratio is remarkably close to the natural semitone identified in Fig. 1 (1.063 ± 0.005), Fig. 2 (1.0600), and in Table 1 (1.045–1.066).

The stretched octave, and the implications for how frequency ratios are discriminated, was investigated in detail by Bonnard et al. (2013). The researchers used both simultaneous octave detection and sequential octave detection, and measured discrimination functions for each of six subjects, which were fundamentally different under the two conditions. Under simultaneous conditions, and making sure that beats were inaudible, the discrimination function peaked precisely at a ratio of 2:1, but for sequential stimuli the discrimination was always a monotonically increasing function of interval size, with no inflection at the octave. Each subject had a unique stretching function and sensitivity to mistuning. The accuracy varied with the individual, and for some the sequential method was more accurate than the simultaneous; for others it was the reverse. On this basis, the paper concluded that there must be two separate “perceptual continua” (perhaps simplicity and magnitude) built in to the ear, and this supports the idea that one of them could be a detector of simple ratios and the other a natural semitone.

#### A musical dimension in auditory space

**Figure 5:**
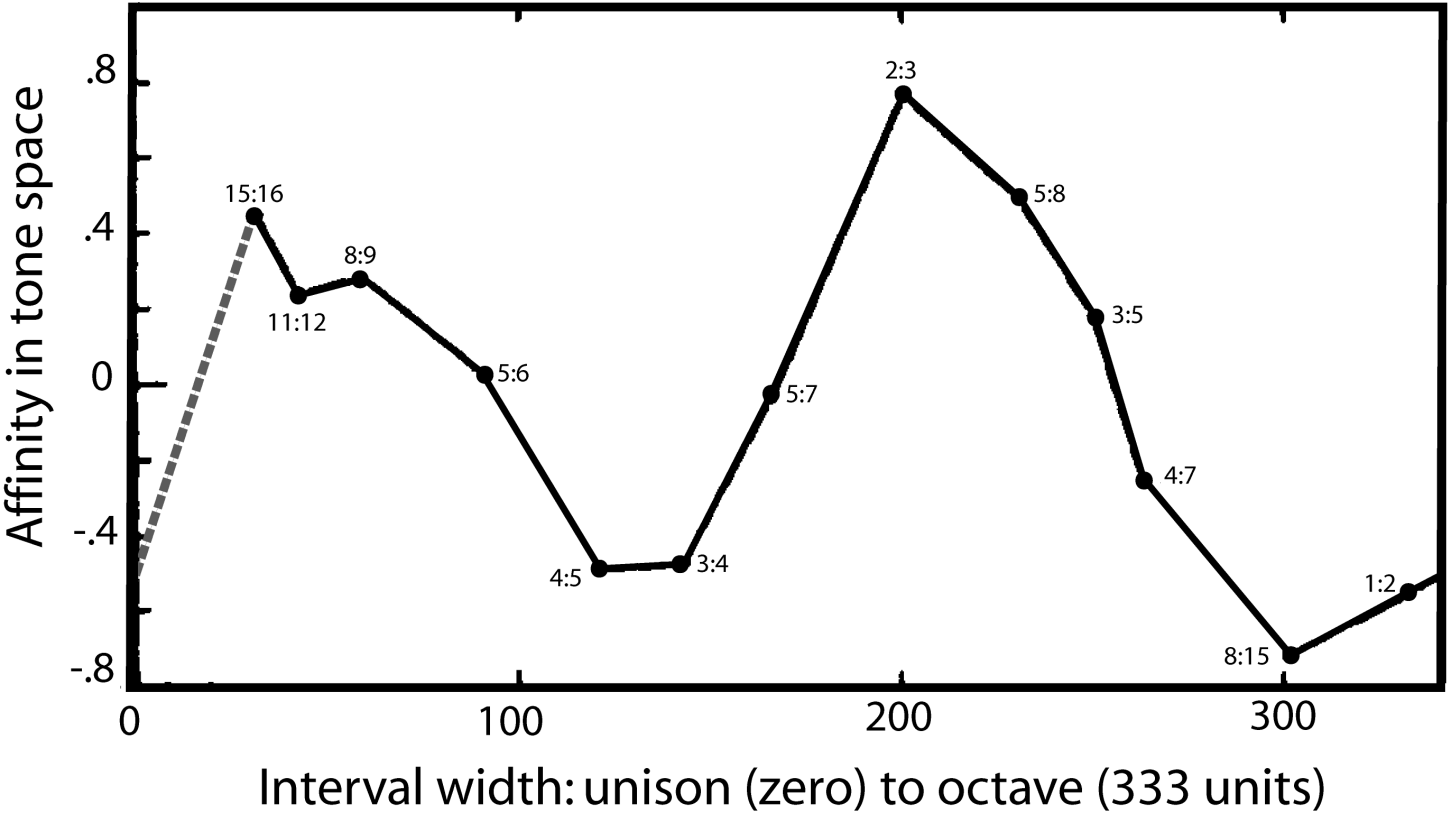
A map of harmonic auditory space as found by Levelt, Van de Geer & Plomp (1966) from presentation of simultaneous tones. In psychophysical experiments, Levelt and colleagues increased the width between two pure tones (*x*-axis) from zero to an octave, and the subjective affinity between the tones, as rated by listeners (*y*-axis), was found to go through two complete cycles, as shown. It shows that the semitone (15:16) and the perfect fifth (2:3) stand out as two auditory landmarks. The dashed grey line is added as an assumed equivalence between the unison and the octave based on their perceptual similarity. Reproduced with permission from John Wiley and Sons. Copyright, John Wiley & Sons, Inc.

Turning to a second strand of evidence, there is one notable study which appears to directly reveal the two-fold nature of musical perception and its fundamental landmarks. The detailed psychophysical investigations of Levelt, Van de Geer & Plomp (1966) found that auditory space—taken to be made up of a continuous range of frequency intervals—could be plotted in a three-dimensional space which allowed four musical intervals to emerge as distinct landmarks in this space—one of which was the semitone. In painstaking experiments, listeners were presented with sets of frequency intervals within the octave and asked to make three-way judgements about which were most similar and which were most dissimilar. Unlike many studies, there was no mention of music, and the criterion used by the subjects was up to them. Using multidimensional scaling, Levelt and colleagues extracted affinities between the ratios, and the findings were that listeners judged the ratios based on three main dimensions. One dimension, of course, was interval width, which ranged from a semitone up to an octave (and beyond). However, Levelt and colleagues found other dimensions in this space which they interpreted as due to the presence of distinct features (or “reference points”). These points caused a peculiar bending in the interval width curve, giving rise to a parabola (or perhaps an ellipse) when the space was mapped onto two of the dimensions. A projection of the parabola onto the primary dimension of interval width is shown in Fig. 5, and the figure clearly shows the musical reference points.

There is the semitone (15:16) at one end of the curve, and the octave (1:2) at the other, but inbetween are two other extrema: an upward peak due to the fifth (2:3) and a downward peak due to the major third (4:5) and perfect fourth (3:4). The authors are puzzled by these extrema and suggest the peaks might be due to a special affinity—a musical sense—surrounding these intervals. In other words, there seems to be an innate ability to pick out certain musical intervals from among a continuum of intervals. One of those is the octave, but the presence of the other peaks suggests the auditory system is able to detect other musical ratios in a similar way, and one of those anchor points includes the semitone.

It would be interesting to repeat this important work, confirm its findings, and fill in some of the missing gaps. The intervals plotted in Fig. 5 are in some places rather sparse, and in particular there is an outstanding gap between the unison (1:1, having zero width) and the semitone (15:16). Theoretically, the unison has much in common with the octave, and is likely to have a similar musical affinity. If it does, the dotted line added to Fig. 5 gives its probable coordinates. The musical loop then becomes closed, and the scale now appears to go through two rises and falls on its progression from the unison to the octave.

As a matter of detail, a closed loop corresponds in Levelt’s paper to fitting an ellipse rather than a parabola to the data, which is actually noted in their paper as giving a better fit. On theoretical grounds, Levelt and colleagues could not see how an extremely narrow interval (e.g., 999:1,000) could end up matching a wide one (e.g., 2:1), but it seems that recognising an equivalence between the unison and the octave would provide a way of completing the cycle. Again, more data would clarify the picture and provide more accurate measures of the locations of the peaks—particularly the semitone, which is predicted to be enlarged (a natural semitone).

### Towards a synthesis

The endpoint of this discussion is to bring together an identified objective property of the cochlea—a peak in OAE frequency spacing of 1.060 ± 0.005—and a subjective preference for one of four musical landmarks in the auditory system (notably the semitone). One possibility is that the semitone is built in to the cochlea as an automatic mechanism to identify melodies. More specifically, the semitone could arise through a step-like organisation of the cochlea’s tuned elements (Fig. 4). The steps may come about either through multiple internal reflection (Shera, 2015) or from innate quantisation of coupled oscillators (Wit & Van Dijk, 2012).

The advantage of the latter model is that it allows melodic intervals to be automatically detected. The coupled oscillator model implies there might be an array of tuned elements in the cochlea, quantised not unlike the way the strings of a harp or piano are. If so, this might give credence to the original Helmholtz model of the cochlea as an array of tuned strings which resonate sympathetically to incoming sound. In fact, there seems to be direct evidence of a regular array of tuned elements—a “keyboard”—in the cochlea. As mentioned earlier, the otoacoustic emissions of subject BD, described by Braun (2013), show regular quantisation, and a closer examination is revealing. Figure 2 of his work shows a run of 10 continuous intervals in the range 1–2 kHz. Reading off values from the semitone scale provided, it can be seen that the “keyboard” from lowest to highest SOAE extends over 10.4 semitones, equivalent to an average of 104 cents per interval. Similarly, there is another run of 11 continuous semitone intervals in the range 2–4 kHz, and here the span is 11.4 semitones, again an average of 104 cents per interval. Such values are very close to the natural semitone interval of 106 cents recorded by us, and justify the assertion that, for this subject at least, there appears to be a well-tuned “natural keyboard” in the cochlea. Note, however, that each individual “key” is not perfectly tuned, so that there is distribution around the mean, as we too have seen.

Against this picture, it is worth noting that Helmholtz himself regarded music—scales, keys, diatonic tunings, and the like—as “the work of artistic invention” and were “by no means furnished by the natural formation or natural function of our ear” (Helmholtz, 1875; pp. 365–6).

There is ample scope for exploring whether the coupled oscillator model can be appropriately applied to the inner ear. One important feature of the coupled oscillator model is that, unlike a stringed instrument, tuning is not static but dynamic: a single tone will entrain a cluster of oscillators on either side of its frequency (Wit & Bell, 2017), and this region of the basilar membrane might provide the benchmark for the perception of subsequent tones. On this basis, if two tones occur concurrently and are separated by a semitone or less, the attempt by the oscillators to entrain to both frequencies at once will fail, and the conflict could well be experienced as a sensation of dissonance. However, if two tones are wider apart than a semitone, and each can entrain its own set of oscillators, there will be no conflict and a perception of harmony could arise.

## Conclusion

This study of a large OAE dataset adds to the literature and confirms that a frequency ratio of 1.06 often occurs in cochlear mechanics. In this study, the distribution clustered around 1.05–1.07, with a peak at 1.063 ± 0.005. It seems unlikely that such a highly significant musical ratio—it is widely used across many (but not all) cultures in some form or another—appears simply by chance, and it may be that the ratio reflects an innate musical property of the cochlea, a possibility that carries major implications for music theory.

In terms of cochlear mechanics, the most common explanation for the 1.06 ratio is in terms of multiple internal reflection, a ‘global’ model with an extensive array of support, both experimental and mathematical. One of its predictions is the generation of a cochlear staircase in which the frequency ratio between the steps is about a semitone. However, the coupled oscillator model also predicts a similar staircase, and more work is needed to decide between them. The advantage of the coupled oscillator model is that it is self-adjusting, so that the steps are formed automatically because of the way oscillators entrain to incoming sound and to each other. This means that each note of a melody could entrain a group of oscillators—a portion of the basilar membrane—to dynamically create a plateau. That is, unlike the multiple internal reflection model, there is no pre-existing staircase (or keyboard); the quantisation only happens in response to an impressed tone. Relative pitch could therefore be extracted by counting steps between neighbouring intervals. Such a mechanism would accord with the “automatic frequency shift detector” described by Demany & Ramos (2005), which is optimally tuned to detect shifts of about a semitone (Demany, Pressnitzer & Semal, 2009).

The coupled oscillator model provides an account of how the semitone could emerge naturally within the cochlea. However, placing this model within the context of music theory will require more development than what has been sketched here (some of the difficulties are laid out in Trainor (2006)). The melodic aspect of music can be accommodated, but fitting it into a complementary harmonic template is a more complex task.

It is suggested that additional psychophysical measurements of the stretched octave, and the magnitude of the ratios producing maximum consonance and dissonance, would be particularly useful, especially if compared with measured ratios between otoacoustic emissions in certain subjects. Detailed investigation of auditory space, along the lines explored by Levelt, Van de Geer & Plomp (1966), would allow fine-grain mapping of internal musical landmarks. In fact, repeating the work using melodic intervals (non-simultaneous tones) would form an important complementary perspective to the original harmonic (simultaneous) work.

Surveying the inherently musical properties of the cochlea could help us understand what is going on in our ears, as well as in our mind, when we listen to music.

##  Supplemental Information

10.7717/peerj.4192/supp-1 Data S1 Data points for Fig. 1 Left column is frequency (kHz); middle column is signal (dB SPL); right column is noise floor (dB SPL).

10.7717/peerj.4192/supp-2 Supplemental Information 1 Frequency ratios between neighbouring SOAEs (left column); lower frequency (Hz) of the pair (right column)

10.7717/peerj.4192/supp-3 Data S2 Data for Fig. 3 Left column is time (seconds); middle column is amplitude (linear units) of SOAE at 2,165.5 Hz; right column is amplitude of SOAE at 2,295.5 Hz.

## Additional Information and Declarations

### Data Availability

The following information was supplied regarding data availability: The raw data has been provided as Supplemental Files.
